# Ac-EAZY! Towards GMP-Compliant Module Syntheses of ^225^Ac-Labeled Peptides for Clinical Application

**DOI:** 10.3390/ph14070652

**Published:** 2021-07-06

**Authors:** Marc Pretze, Falk Kunkel, Roswitha Runge, Robert Freudenberg, Anja Braune, Holger Hartmann, Uwe Schwarz, Claudia Brogsitter, Jörg Kotzerke

**Affiliations:** 1 Department of Nuclear Medicine, University Hospital Carl Gustav Carus, Technical University Dresden, 01307 Dresden, Germany; roswitha.runge@ukdd.de (R.R.); robert.freudenberg@ukdd.de (R.F.); anja.braune@ukdd.de (A.B.); holger.hartmann@ukdd.de (H.H.); claudia.brogsitter@ukdd.de (C.B.); 2 Molecular Imaging and Radiochemistry, Department of Radiology and Nuclear Medicine, Medical Faculty Mannheim, Heidelberg University, 68167 Mannheim, Germany; 3 Eckert & Ziegler Eurotope, 13125 Berlin, Germany; Falk.Kunkel@ezag.de; 4 Eckert & Ziegler Radiopharma, 38110 Braunschweig, Germany; Uwe.Schwarz@ezag.de

**Keywords:** actinium-225, TATE, PSMA, module synthesis, endoradiotherapy, GMP

## Abstract

The application of ^225^Ac (half-life T_1/2_ = 9.92 d) dramatically reduces the activity used for peptide receptor radionuclide therapy by a factor of 1000 in comparison to ^90^Y, ^177^Lu or ^188^Re while maintaining the therapeutic outcome. Additionally, the range of alpha particles of ^225^Ac and its daughter nuclides in tissue is much lower (47–85 μm for alpha energies E_α_ = 5.8–8.4 MeV), which results in a very precise dose deposition within the tumor. DOTA-conjugated commercially available peptides used for endoradiotherapy, which can readily be labeled with ^177^Lu or ^90^Y, can also accommodate ^225^Ac. The benefits are lower doses in normal tissue for the patient, dose reduction of the employees and environment and less shielding material. The low availability of ^225^Ac activity is preventing its application in clinical practice. Overcoming this barrier would open a broad field of ^225^Ac therapy. Independent which production pathway of ^225^Ac proves the most feasible, the use of automated synthesis and feasible and reproducible patient doses are needed. The Modular-Lab EAZY is one example of a GMP-compliant system, and the cassettes used for synthesis are small. Therefore, also the waste after the synthesis can be minimized. In this work, two different automated setups with different purification systems are presented. In its final configuration, three masterbatches were performed on the ML EAZY for DOTA-TATE and PSMA-I&T, respectively, fulfilling all quality criteria with final radiochemical yields of 80–90% for the ^225^Ac-labeled peptides.

## 1. Introduction

Targeted alpha therapy (TAT) is a promising approach for the treatment of cancer [1]. The use of alpha emitters for cancer therapy has three distinct advantages over conventional therapies with beta emitters: The short range of alpha radiation in human tissue (less than 0.1 mm), corresponding to only a few cell diameters, allows the selective killing of targeted cancer cells while sparing surrounding healthy tissue. At the same time, the high energy (several MeV) of alpha particles and its associated high linear energy transfer leads to a high rate of cell deaths. Consequently, alpha radiation can destroy cells, which otherwise exhibit resistance to treatment with beta or gamma irradiation or chemotherapeutic drugs, and thus can offer a therapeutic option for tumors resistant to conventional therapies. The third is the radiation safety for personnel as ^225^Ac-therapeutic doses are in the MBq range (~100 kB/kg) compared to several GBq used commonly for ^90^Y- and ^177^Lu-therapy. Recent results demonstrating the remarkable therapeutic efficacy of alpha emitters to treat various cancers have underlined the clinical potential of TAT. To date, the chelator DOTA is commonly used for ^225^Ac-labeling of peptides, antibodies and small molecules [2,3,4] together with the well-known diagnostic partner nuclide ^68^Ga (half-life T_1/2_ = 68 min). Therefore, well-known diagnostic radiotracers, such as TATE, PSMA or RGD are available for ^225^Ac-labeling and therapy. Additionally, the quality control for clinical routine production is a complex process because of the daughter nuclides of ^225^Ac [5,6]. Further, the three photopeaks of ^225^Ac (78 keV), ^221^Fr (218 keV) and ^213^Bi (440 keV) can be measured by SPECT [7], or the Cerenkov radiation of ^213^Bi can be used for Cerenkov luminescence imaging [4,8] for therapy control and dosimetry.

As the great potential of targeted cancer treatment with alpha emitters is beginning to draw worldwide attention [9,10,11,12], the demand for the radionuclide ^225^Ac is expected to increase by several orders of magnitude. The current production via chemical separation from existing stocks of ^229^Th cannot meet the projected demand [13]. Consequently, a variety of alternative production routes based on the irradiation of uranium, thorium or radium targets at reactors or accelerator facilities are being investigated, and a combination of different sources will likely be required to meet the growing demand [14]. Among the new production methods, the irradiation of ^226^Ra by medium energy protons or by neutrons shows great promise, but significant challenges involving the handling of radium targets must be surmounted [15].

Once the supply with ^225^Ac is secured by new methods or a combination of several methods, safe and reliable synthesis methods have to be developed to minimize waste production and protect the environment and operator against contamination. Automated synthesis systems are ideal for GMP-compliant production in controlled and closed environments. Several automated systems for example from Eckert & Ziegler [16,17], Elysia-Raytest [18], IBA molecular [19], iPHASE [20], Scintomics [21,22] and Trasis [23] are currently available for diverse GMP-compliant diagnostic and therapeutic radiotracer production.

The aim of this work was to evaluate the efficiency and reliability of the radiosynthesis of ^225^Ac-labeled DOTA-conjugated peptides and establish the translation of the synthesis to an automated synthesis platform (Modular-Lab EAZY, Eckert & Ziegler) for clinical routine production [24]. The ML EAZY is a cassette-based module operated with GMP-compliant software [25]. Two different SPE purification methods by C18- and CM-cartridges were compared. After careful validation of the process, the ^225^Ac-labeled peptides are now available in radiochemical yields (RCYs) of 80–90% for tumor therapy in patients in accordance with the regulations of the German Pharmaceuticals Act §13.2b.

## 2. Results

### 2.1. Manual Evaluation of ^225^Ac from Two Different Sources for Radiolabeling of DOTA-Conjugated Peptides

For an adequate translation from manual synthesis to automated clinical routine production, the following test parameters were chosen:

- Buffer constitution;
- Buffer volume (1–4 mL);
- Buffer pH (5.2–6.8);
- Reaction pH (4.8–5.8);
- Reaction temperature (90–105 °C);
- Reaction time (25–40 min);
- Precursor concentration (10–50 µg/MBq);
- Activity amount (0.5–18 MBq);
- Activity matrix and volume (water, 0.04 M HCl, 0.1 M HCl);
- C18 cartridge purification;
- CM cartridge purification;
- Endotoxin level;
- Sterility;
- The influence of the time of quality control on the result (sampling time after EOS, time between removal of TLC plate from tank to analysis)

A reaction pH of 5.0–5.5 should be maintained for the efficient complexation of ^225^Ac to DOTA. Higher pH leads to the formation of insoluble ^225^Ac-hydroxide, while lower pH leads to lower complexation.

The following buffers were tested:

1. Sodium ascorbate (0.1 M): Even though 2 mL of sodium ascorbate (0.1 M) buffer resulted in a high yield (>90% RCY) of ^225^Ac-labeled peptides, some drawbacks were observed: the buffer is not stable longer than 1 month at −20 °C, leading to a significant decrease of RCY with time. The volume of 2 mL of this buffer is only capable of buffering 0.1 mL HCl (0.04 M). When activity is delivered in a larger volume than 0.1 mL, the buffer volume has to be increased accordingly to keep control of the reaction pH. The increased volume may exceed the maximal volume capacity of the delivery vial when desired to use it as a reaction vial.
2. Sodium acetate (0.9 M): The EZ-102 kit contains three vials: Vial 1: sodium acetate trihydrate (680 mg); Vial 2: H_2_O (3.6 mL); Vial 3 0.96 M acetic acid and 0.7% HCl. To prepare a 0.9 M solution, 680 mg of the sodium acetate trihydrate are dissolved completely in 3.6 mL of water from the kit. The pH is adjusted to pH between 5.0–5.5 by the addition of acetic acid. This buffer tolerates higher volumes of HCl while maintaining the pH between 5.0–5.5. The disadvantage to this buffer is that the higher molarity leads to a much lower complexation <10% RCY.
3. Sodium acetate (0.1 M): In total, 0.15 mL of acetic acid was added to a 0.9 M sodium acetate solution, and the mixture was diluted with H_2_O by factor 9 to obtain 0.1 M sodium acetate/acetic acid buffer with a pH of 5.7–5.8. By adding different volumes (0.1 or 0.5 mL) of 0.04 M HCl, the resulting reaction pH is between 5.5–5.0, respectively. This buffer can also be stored at −20 °C for at least two months. The 0.1 M sodium acetate/acetic acid buffer was regarded as the best buffer, and the three tests resulted in RCYs 80–90%.

When the ^225^Ac (~3% γ-coemission between 60 keV and 100 keV) activity is delivered, all the main daughter nuclides, namely ^221^Fr (12% γ-coemission 218 keV), ^217^At and ^213^Bi (26% γ-coemission 440 keV) are in an equilibrium state. A complete decay chain of ^225^Ac and its daughter nuclides with modes of decay and energies can be found in Table S2. The dose calibrator (ISOMED 2010, NUVIA instruments, Germany) was calibrated by a certain chamber factor from the fabricator for the equilibrium state, which corresponds to the correct activity or starting activity. The purification of the reaction solution by C18- or CM-cartridges leads to differing separation states of the mother and daughter nuclides and in consequence to a time-dependent quality control result. Otherwise, the activity would have to be measured with a new chamber factor at the end of synthesis. Hence, using the same factor as for the starting activity, the correct activity can only be measured when the daughter nuclides are in an equilibrium state again. After a time >2 h, the measured activity corresponds to >97% of the activity in an equilibrium state.

With purification by C18 cartridge, mainly free ^221^Fr and ^213^Bi are directed into the waste vial and show >25% of the starting activity, while the ^225^Ac-labeled peptide in the product vial exhibits <75% of the starting activity in equilibrium. After 20–40 min (4–8 half-lives of ^221^Fr), the measured activity in the waste vial decreases below 10% of the starting activity, while the activity in the product vial increases above 90% of the starting activity. After 12 h (10 half-lives of ^213^Bi), less than 3% of the starting activity is left in the waste vial, while the product vial contains more than 97% of the starting activity.

For purification with the CM cartridge, mainly free ^213^Bi and ^225^Ac are trapped, while ^225^Ac-labeled peptide and free ^221^Fr are transferred into the product vial resulting in disruption of the equilibrium and challenges of the activity determination. First, the CM cartridge was tested for the trapping efficiency of free ^225^Ac in solution. 1.0 MBq was diluted with 5 mL 0.9% NaCl and eluted through the CM cartridge. 2 h after elution, 1.0 MBq of ^225^Ac was measured on the CM cartridge, indicating complete trapping of free ^225^Ac. Interestingly, ^221^Fr was eluted from the CM-cartridge by 2 mL saline, while the ^225^Ac and ^213^Bi remained on the cartridge. It was also tested whether it is possible to elute only ^213^Bi from the cartridge by either 1 mL HCl (0.1 M) or a 1-mL-mixture of HCl (0.05 M) and NaI (0.05 M) [26]. However, this experiment resulted in nearly complete elution of ^225^Ac, while certainly, ^213^Bi remained on the CM cartridge as the measured residual activity on the CM cartridge halved every 40 min. To evaluate the trapping efficiency of ^225^Ac and its daughter nuclides on CM cartridge, 2.0 MBq ^225^Ac in equilibrium state was mixed together with 2.1 MBq purified ^225^Ac-labeled peptide, and the mixture was passed through a new CM cartridge (Table 1). Interestingly, <75% of the correct activity was measured on the CM cartridge (free ^225^Ac, ^213^Bi) right after purification, while the additional free ^221^Fr in the product vial led to >133% of the correct activity. After 20–40 min (4–8 half-lives of ^221^Fr), the activity on the CM cartridge increased to >85% of the correct activity, while the activity of the product vial decreased to >115% of the correct activity. Sixty minutes after purification, the additional free ^221^Fr in the product vial was decayed, and on the CM cartridge, the equilibrium nearly restored, leading to a lower difference of 5% to the correct activity. After 16 h >99% of the expected (real) activities were restored both in the product vial and on the CM cartridge (20 half-lives of ^213^Bi).

Therefore, the activity of the product should always be measured after the quality control (30–40 min) to detect >90% of the correct activity, regardless of whether C18 or CM cartridges were used for purification. However, the exact volume activity can already be measured by gamma spectrometry of a defined volume (HPGe detector, e.g., 100 µL) of the product and integration of the ^225^Ac peak at 78 keV right after purification and formulation.

### 2.2. Transfer of the Manual Process to Modular-Lab EAZY

For the adaption to the Modular-Lab EAZY, the following conditions were considered: To use ^225^Ac-chloride and ^225^Ac-nitrate from the different suppliers, the reaction time must be at least 35 min although ^225^Ac-chloride required only 25 min for complete complexation with the amount of precursor per activity at 20 µg/MBq for both PSMA and TATE peptides, since the complexation rate of ^225^Ac-nitrate to DOTA was found to be lower, which is consistent with other nuclides such as ^68^Ga or ^177^Lu. Even though the optimal temperature for the reaction is 90 °C, the setpoint for the reaction had to be increased to 105 °C to achieve optimal RCY.

The EAZY-Ac-Peptide-cassette for C18 purification consists of a modified standard cassette for labeling DOTA peptides with Ga-68 (Figure S6). The reactor was replaced by a reactor from a C0-LUDOTAPEP-CM standard cassette. The buffer and eluent vials are conic with a micropin hole (MP1000, B.Braun, Maria Enzersdorf, Austria). Buffer and eluent were added through these micropin holes by syringe and cannula. For venting purposes, the cannula should only be situated halfway through the micropin holes. The C18 cartridge was activated by 1 mL EtOH, followed by 2 mL H_2_O and was connected in wet status. The cassette for CM purification consisted of the C0-LUDOTAPEP-CM standard cassette (Figure S7). The CM cartridge was activated by 3 mL H_2_O and was connected in dry status for lower volume in the product vial. A Sterican cannula (4665791, B.Braun) may be used for transfer of liquids and activity, as they are silicon-coated, ensuring low metal input into the reaction solution. A filter system of two ultra-low protein-binding sterile filters (vented SLGVV255F followed by SLGV033R) was used for filtration since the loss of the product on this type of filter is <5% for both together. Using two filters has the benefit of double-safety in case one of the filters is damaged. The vented filter serves as a “filter-integrity-test” since no pressure higher than 3.6 bar can be reached (both Ac-EAZY methods work with a maximum of 1.4 bar). A complete transfer is achieved by this configuration as it tolerates intermediate gas flow through the vented filter. For a detailed reaction setting and outcome, see supporting information.

### 2.3. Validation of the Automated Syntheses with C18 or CM Purification

**Figure 1:**
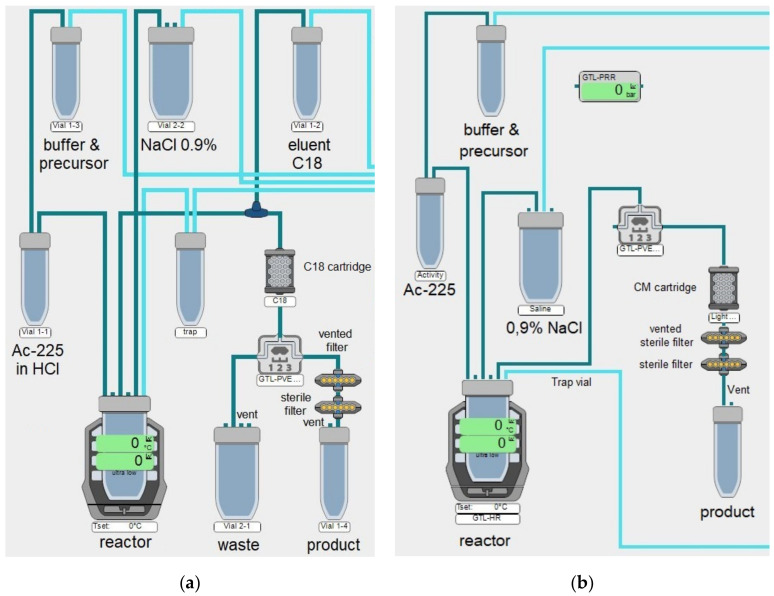
A schematic depiction of the synthesis scheme of the Modular-Lab EAZY module: (**a**) the C18 method; (**b**) the CM method.

Both purification methods were transferred to the Modular-Lab EAZY and were tested for reproducibility, stability and transfer for routine production, and the module was assembled as depicted in Figure 1. After 30 syntheses, an optimal peptide concentration of 20 µg/MBq was found. A complete reaction overview can be found in Table S1 and Figure S8. For purified ^225^Ac from ITM, also lower concentrations of 10 µg/MBq are possible for RCY of 80–90%. Three validation batches were performed for each ^225^Ac-DOTA-TATE and ^225^Ac-PSMA-I&T to prove the reproducibility of the automated syntheses. The following acceptance criteria were used to decide for a successful automated production:

- RCP >80% prospective, >95% retrospective (if activity is >80% prospective, a retrospective measurement will be >99% for silica gel on aluminum with citrate)
- Endotoxin level <5.00 EU/mL
- RCY 80–90%
- Product pH 4.0–8.0

## 3. Discussion

Based on the data of 45 manual syntheses, the following conclusions can be drawn:

The buffer capacity of sodium ascorbate is too low, and it cannot be stored for more than a month at −20 °C. If the volume of ^225^Ac is higher than 0.1 mL, then the volume of the sodium ascorbate buffer must also be increased.

The buffer capacity of acetate buffer prepared from the EZ-102 reagent set was high enough for using 0.1–0.5 mL HCl (0.04 M), which is used for ^225^Ac delivery. Dilution from 0.9 M to 0.1 M increased the RCY when using this buffer system. Additionally, it can be stored at −20 °C for more than 3 months.

After the synthesis, around 5% of starting activity was found in the activity vial (KIMAX), although it was rinsed with 2 mL of the buffer solution. Additionally, around 5% of starting activity was found in the reactor, although it was rinsed with 3 × 2 mL saline. Further, around 5% activity was found on the purification cartridges and the two filters. This leads to a loss of starting activity of ~15% for the whole process. Therefore, an RCY of 80–90% is reasonable.

A certain discrepancy in the quality (specific activity) of the different ^225^Ac-sources was found. ^225^Ac-chloride from ITM is purified one day prior to shipping. Therefore, precursor concentrations of 10 µg/MBq were sufficient to obtain RCYs >80%. ^225^Ac-chloride could be used for one week without a significant decrease in RCYs. ^225^Ac-nitrate was delivered within one week from Obninsk in solid form and was dissolved on-side with 0.04 M HCl avoiding any contamination by metal ions. However, precursor concentrations of 20–30 µg/MBq were necessary to obtain RCYs >80%, depending on batch and storage time of the activity. ^225^Ac-nitrate yielded lower RCYs after two weeks and therefore should not be used if it is older than one week. Only a higher concentration of 50 µg/MBq delivered RCYs >80% after two weeks, presumably as a consequence of ^209^Bi enrichment, which is a competitor in the ^225^Ac-radiolabeling. After three weeks of storage of the ^225^Ac-solution, even that high concentration of precursor delivered RCYs of only <10%.

However, DOTA is not the ideal chelator for ^225^Ac because of the long labeling time, high temperature, and the need for high molar amounts of precursor (10–20 µg/MBq) and, as a consequence, better chelators are currently under development [27,28,29,30,31,32]. For example, the chelator macropa forms stable 2^25^Ac-complexes within 5 min at room temperature and at lower precursor concentrations. The positron-emitting ^132^La (T_1/2_ = 4.6 h) is being discussed as the diagnostic partner nuclide [27].

The high amount of peptide (20 µg/MBq) results in a maximum dose of 200 µg/10 MBq ^225^Ac-labeled peptide, which is equal to the amount used for routine ^177^Lu-preparations with 8000 MBq [33]. Typically, a single dose of 6–8 MBq is administered per patient, containing 120–160 µg peptide. The necessity to use high amounts of precursor leads to complexation not only of ^225^Ac but also likely of its daughter nuclides ^213^Bi and ^209^Pb, which can be exploited for quality control. If the quality control sample is removed quickly from the final product and immediately submitted to TLC, the waiting time for the correct result was reduced to <30 min since the amount of free ^213^Bi is low. During the reaction, any ^213^Bi generated by free radionuclides released by recoil from the chelator was a rebound. In the final solution, 50% free ^213^Bi prolonged the waiting time for correct RCP values to >120 min (Figures S1–S5). The TLC to test for free ^225^Ac in citrate buffer must be performed on silicagel–aluminum since both ^225^Ac-PSMA (Figure S3) and ^225^Ac-TATE (Figure S5) migrate with the front together with free ^225^Ac on ITLC-SG. The TLC for colloidal ^225^Ac-hydroxide in NH_4_Ac:MeOH can be performed on ITLC-SG for ^225^Ac-TATE (Figure S4). Interestingly, the TLC for colloidal ^225^Ac-hydroxide for ^225^Ac-PSMA-I&T must be performed on silicagel–aluminum as well (Figure S1) since it migrates as a very broad peak on the ITLC-SG (not shown).

Following the literature, DTPA (~0.1 mg/mL) is frequently added to the product for the complexation of free daughter nuclides and fast renal excretion [10], as well as ascorbic acid for preventing radiolysis [10]. DTPA can either be dissolved in the saline, in the eluent or in the product vial. It was evaluated whether it is possible to use the eluent from EZ-102-V2 (H_2_O:EtOH 1:1) and mix it in a second flask containing the sterile DTPA (1.2 mg in 1.5 mL = 0.8 mg/mL, diluted with >7 mL saline reach a final concentration of DTPA of ~0.1 mg/mL), and this method worked reliably. However, despite the literature, DTPA may be excluded from the final formulation if a purification method such as C18 or CM cartridges is used to remove non-chelated ^225^Ac. Nonetheless, free ^213^Bi is removed fast by renal elimination [34,35]. However, since a higher amount of precursor peptide (20 µg/MBq) resulted in the complexation of ^225^Ac and ^213^Bi (and other daughter nuclides), DTPA is considered no longer necessary in the final formulation and was therefore eliminated from the synthesis for the sake of viability.

The stability of the final ^225^Ac-labeled peptides was also tested, since the radiolysis and alpha decay is often discussed as fatal for the radioligands [36]. Therefore, the ^225^Ac-labeled peptides remained in the product vial for 24 h at room temperature after the synthesis without DTPA or ascorbic acid before sampling. Right after development, 50% of activity was found as unbound activity on the TLC chromatogram. However, the activity of the spot for unbound radionuclides decreased over time and >5 h after development, the RCP was again >95%, indicating no significant loss of ^225^Ac from the complex. This is an important fact, which could lead to central production sides of ^225^Ac-labeled peptides in future. Presumably, the low activity (5–8 MBq) and long half-life of ^225^Ac leads to the observed low radiolysis of the peptides. Maybe an additional cartridge purification after shipping would be necessary in that case.

## 4. Materials and Methods

The precursor for ^225^Ac-DOTATATE (DOT05/02/20) was obtained from ROTOP (Dresden-Rossendorf, Germany). The precursor for ^225^Ac-PSMA-I&T (CAO-191006/01) was obtained from piChem (Grambach, Austria). All other reagents and solvents were purchased in the highest available purity from Merck (Darmstadt, Germany). Reagent kits for cassette assembly were obtained from E&Z. SepPak C18 light (WAT023501) and CM light (WAT023531) cartridges were purchased from Waters (Milford, MA, USA). The Modular-Lab EAZY module (GTL) with software Modular-Lab v6.2 was obtained from Eckert & Ziegler (Berlin, Germany). Solvents for quality control were stored at 4 °C. Buffer and precursor were stored at −20 °C; other chemicals were stored at room temperature. The dose calibrator (calibrated by a Cs-137 source AN-1426) and the CoMo-170 for separate α-detection and β/γ-detection were obtained from NUVIA Instruments. The pH was acquired by a QuantoFix Relax reflection photometer (91346) with the corresponding pH test strips 5.5 × 85 mm pH-Fix 2.0-9.0 (92118) (Macherey Nagel, Feucht, Germany). ITLC-SG strips (SGI0001) were obtained from Agilent and silica gel on aluminum strips from Merck. The TLC scanner MiniScanPRO+ was provided by E&Z, the HPGe detector GC2018 was purchased from Canberra (Rüsselsheim, Germany), and the endotoxin test device, EndoSafe, was obtained from Charles River (Sulzfeld, Germany).

The costs for the automated synthesis can be estimated as follows: the peptides would cost €600–1000, the cassettes would cost €180–200, and the ML EAZY would cost €~30,000. To date, 1 MBq ^225^Ac costs around €300–390.

### 4.1. Manual Synthesis of ^225^Ac–Peptides

- Peptide to buffer in a syringe, attach blue micropin (MP1000 B.Braun) to the syringe;
- Peptide–buffer mixture to ^225^Ac in KIMAX vial, shake gently;
- Reaction of 40 min at 90 °C heating block;
- 5 min cooling, venting, dilute with 2 mL saline into 5-mL-syringe.

### 4.2. C18 Purification Method

- The C18 cartridge was rinsed with by 1 mL EtOH, followed by 2 mL H_2_O;
- The diluted reaction solution onto C18 with the 5-mL-syringe into waste;
- Rinse reactor with 2 mL of saline, then rinse onto C18 into waste;
- Elute product with 1 mL 70% EtOH from C18 over a sterile filter into the product vial;
- Rinse C18 with 7 mL DTPA-containing saline over a sterile filter into the product vial.

### 4.3. CM Purification Method

- The CM cartridge was rinsed with 3 mL of H_2_O;
- The diluted reaction solution onto CM with a 5-mL-syringe over a sterile filter into the product vial;
- Rinse reactor with 2 mL of saline, then pour the CM into the product vial;
- Second rinsing of reactor with 2 mL of saline, then pour the CM into product vial.

### 4.4. Quality Control

- RCP was determined by TLC either by cutting the developed TLC in two pieces and measuring their activity by CoMo-170 or by TLC scanner using a beta-sensitive probe.
- For free ^225^Ac, TLC was performed on silica-gel–aluminum sheets in 0.1 M citrate buffer (pH 5.0).
- For colloidal ^225^Ac-hydroxide, TLC was performed in 1 M NH_4_Ac:MeOH 1:1 on silica-gel–aluminum sheets for 225Ac-PSMA-I&T and on ITLC-SG for 225Ac-TATE.
- Radionulidic purity and the exact volume activity were determined by an HPGe detector.
- In total, 10 µL of the product solution was diluted with 990 µL of sterile water and used for the determination of the endotoxin level with EndoSafe.
- The pH was determined with the pH meter Quantofix.

## 5. Conclusions

In summary, the reaction conditions for ^225^Ac-labeling from different sources of the radionuclide were optimized, and two different purification methods were compared and tested for automatization. The Ac-EAZY-peptide cassette for C18 purification consists of a standard cassette, and the reactor was changed by the reactor of a LuPep standard cassette. The Ac-EAZY-peptide cassette for CM purification consists of a slightly modified single LuPep cassette. The CM method seemed superior since no liquid waste was produced throughout the synthesis and no EtOH-containing eluent for C18 cartridge elution had to be used. Considering routine production, the CM method involves two preparation steps less, in brief, the addition of eluent to the corresponding vial and the addition of EtOH to the reactor for automated C18 conditioning. For the use of ^225^Ac from different sources with one cassette, the concentration of precursor per activity of 20 µg/MBq was identified for both PSMA and TATE peptides, and the reaction time should be at least 35 min at 105 °C. Finally, both methods stably yielded ^225^Ac-labeled peptides with RCYs of 80–90% after 48–50 min and RCPs >95%. A reliable and safe method to produce ^225^Ac-products avoiding the production of liquid waste, which can be carried out in closed compartments to avoid the release of activity into the environment, was described. The contaminated single-use cassette can be disposed of after the synthesis.

## Figures and Tables

**Table 1:** Manual testing of trapping efficiency of the CM cartridge by a mixture of 2.0 MBq free ^225^Ac and 2.1 MBq ^225^Ac-labeled peptide by comparison of the measured activities and radiochemical purity (RCP).

Measuring Time after Elution	1 min	40 min	60 min	16 h
activity cartridge (MBq)	1.5	1.7	2.1	2.0
%RCP of the correct activity	75	85	95	>99
activity eluent (MBq)	2.8	2.3	2.2	2.0
%RCP of the correct activity	133	115	105	>99

## Data Availability

Data is contained within the article.

## Supplementary Materials

The following are available online at https://www.mdpi.com/article/10.3390/ph14070652/s1, A detailed step-by-step description of the automated processes. Figures S1–S5: Analytical radio-TLC scans, Figure S6: Modular-Lab EAZY module in its final configuration for C18 purification, Figure S7: Modular-Lab EAZY module in its final configuration for CM purification, Figure S8: Graphical illustration for Table S1 of RCYs, Table S1: Overview of ^225^Ac-syntheses on ML EAZY, Table S2: Decay chain of ^225^Ac and its daughter nuclides.
