# Supplementary material for: Ac-EAZY! Towards GMP-Compliant Module Syntheses of 225Ac-Labeled Peptides for Clinical Application
Source: Pharmaceuticals (Basel). 2021 Jul 6;14(7):652. doi: 10.3390/ph14070652 (PMC8308848; doi:10.3390/ph14070652)
Supplement: Supplementary file 1 [file pharmaceuticals-14-00652-s001.zip › pharmaceuticals-1256629-supplementary.pdf]

Ac-EAZY! Towards GMP-compliant module syntheses of ^{225}Ac-labeled peptides for clinical application

M. Pretze[1,2*], F. Kunkel[3], R. Runge[1], R. Freudenberg[1], A. Braune[1], H. Hartmann[1], U. Schwarz[4], C. Brogsitter[1] and J. Kotzerke[1*]

1 Department of Nuclear Medicine, University Hospital Carl Gustav Carus, Technical University Dresden, 01307 Dresden, Germany

2 Molecular Imaging and Radiochemistry, Department of Clinical Radiology and Nuclear Medicine, Medical Faculty Mannheim of Heidelberg University, 68167 Mannheim, Germany

3 Eckert & Ziegler Eurotope, Berlin, Germany

4 Eckert & Ziegler Radiopharma, Braunschweig, Germany

Supporting information

Step-by-step description of the automated ^{225}Ac-peptide synthesis

A total of 6–18 MBq ^{225}Ac-salt in 100–500 µL 0.04–0.1 M HCl was obtained from ITM (Garching, Germany) or E&Z (Braunschweig, Germany) as starting radioactivity in a KIMAX vial.

- For C18 method only: Attach C18 cartridge to the cassette and add 2 mL $EtOH_{absolute}$ to reactor for automated C18 conditioning.
- For CM method only: Rinse CM cartridge with 3 mL $H_2O_{suprapur}$ and attach in dry state to the cassette.
- Mix peptide with buffer in syringe; attach short cannula (B.Braun 4657667).

- Remove blue micropin (B.Braun MP1000) from gold-capped vial and add buffer from syringe, attach micropin.
- For C18 method only: Remove blue micropin from red-capped vial and add eluent, attach micropin.
- Attach two sterile filters to a vented product vial and connect with product line.
- Attach both green lines to saline completely with a long cannula (B.Braun 4665791) to the reactor and a short cannula (B.Braun 4657667) coming from the cassette.
- Attach both red lines to KIMAX vial (contains activity) completely with a long cannula (B.Braun 4665791) to the reactor and a short cannula (B.Braun 4657667) coming from the buffer vial.

1st minute: the reactor is preheated to 50°C for 2 minutes.

3rd minute: ^{225}Ac-salt is transported into reaction vial and the activity vial is simultaneously rinsed with 2.1 mL buffer-precursor mixture into the reaction vial.

4th minute: the reaction at 105°C for 35 minutes.

39th minute: the reactor is switched off for 5 minutes to cool.

43rd minute: 2 mL of saline is added into the reactor and the diluted reaction solution is transferred through the cartridge.

44th minute: 2 mL of saline is added into the reactor to remove residual activity and the solution is transferred through the cartridge.

45th minute: 2 mL of saline is added into the reactor and to remove residual activity and the solution is transferred to the cartridge.

46th minute: the residual reactor content is completely transferred through the CM cartridge and two sterile filters into the product vial with transfer pressure 1.4 bar.

(For C18: 46th minute: C18 cartridge is eluted with 1.5 mL eluent through two sterile filters into the product vial.

48th minute: 7.5 mL saline are added through the reactor for dilution of the product.

49th minute: the saline from the reactor is completely transferred through the C18 and the two sterile filter into the product vial with transfer pressure 1.4 bar).

48th (50th) minute: the final ^{225}Ac-labeled peptide is colorless and is ready for injection in a volume of 8 mL (CM method) or 10 mL (C18 method), pH 6.0 ± 0.2 and an endotoxin level <5.0 EU/mL. For C18 method, the content of EtOH in the final injection solution is 7.5%. After 48–50 min synthesis time the RCYs are 80–90% with RCPs >95%. The dose for the personnel was 1–2 μSv in 1 h, which accounts mostly for the normal background radiation, regardless of manual or automated synthesis.

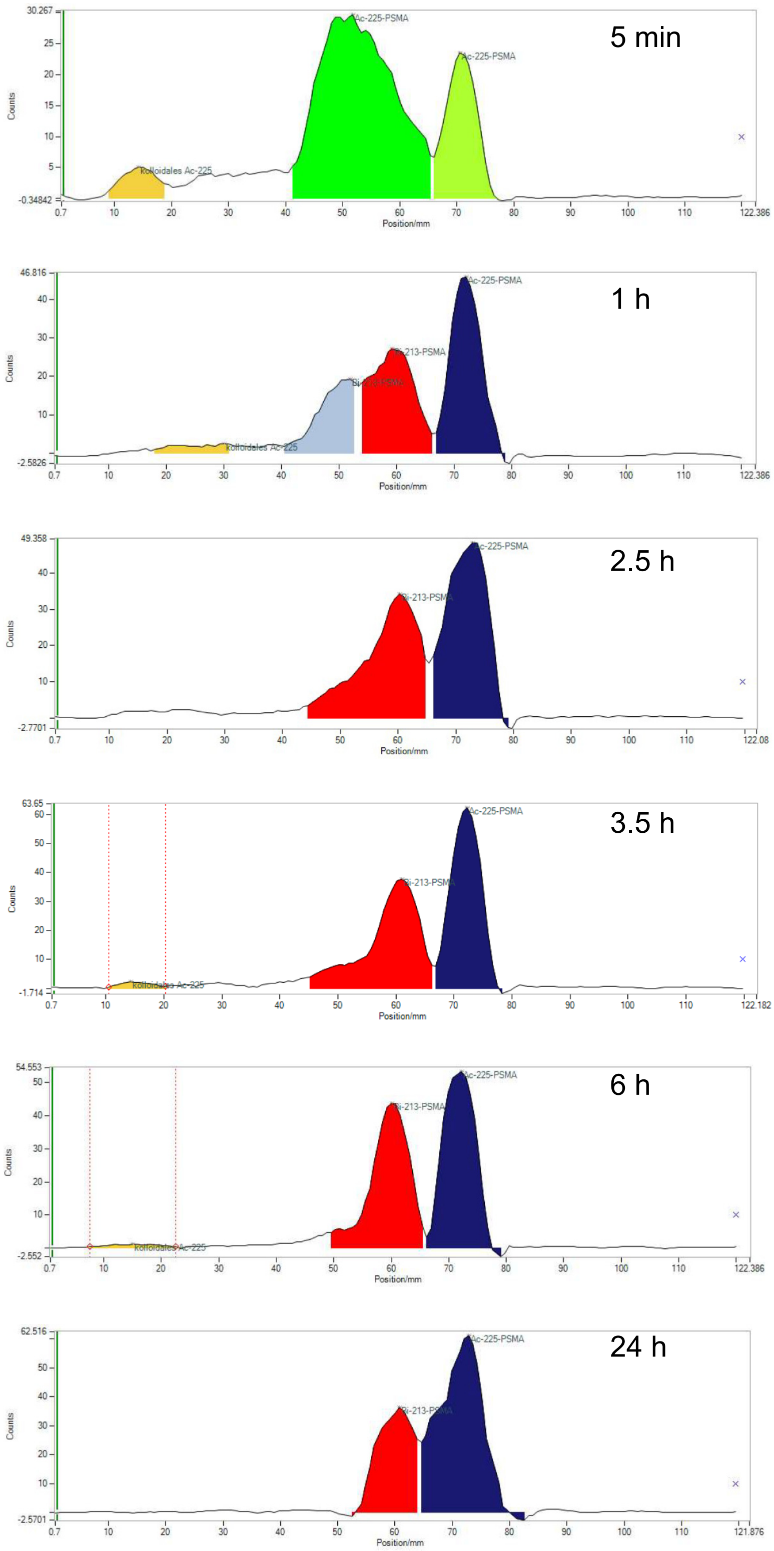

Figure S1. Analytical radio-TLC of ^{225}Ac-PSMA-I&T 1 M NH_4Ac:MeOH 1:1 on ITLC-SG). From top to bottom: 5 min, 1 h, 2.5 h, 3.5 h, 6 h and 24 h same plate scanned past TLC development. Colloidal ^{225}Ac <4% 1 h past TLC development.

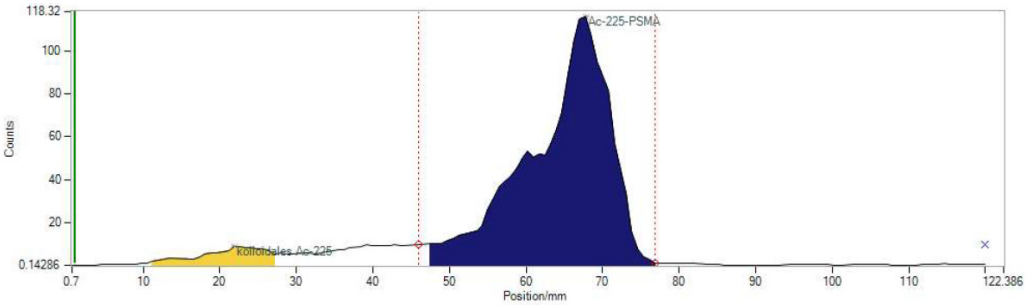

Figure S2: Analytical radio-TLC of another batch of ^{225}Ac-PSMA-I&T 1 M NH_4Ac:MeOH 1:1 on ITLC-SG) with lesser double peak formation. Colloidal ^{225}Ac <6% 10 min past TLC development.

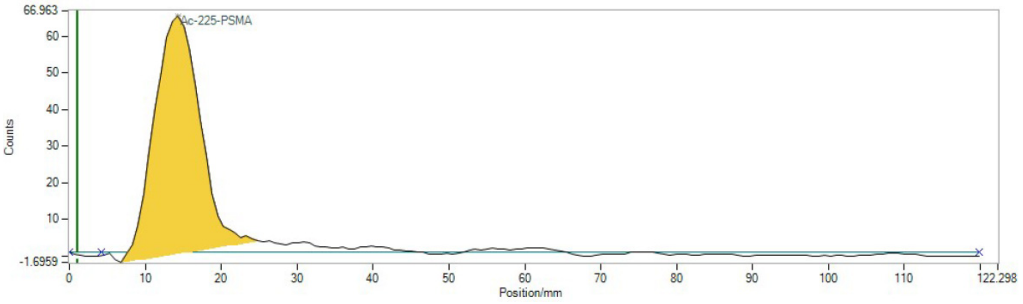

Figure S3: Analytical radio-TLC of ^{225}Ac-PSMA-I&T on silica gel-aluminum 60 Å F254. RCP >99% 1 h past TLC development.

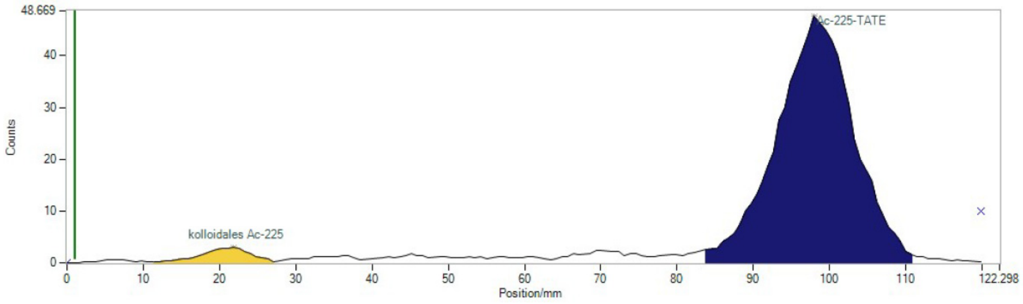

Figure S4: Analytical radio-TLC of ^{225}Ac-TATE after purification using a CM cartridge (1 M NH_4Ac:MeOH 1:1 on ITLC-SG). Colloidal ^{225}Ac <4% 1 h past TLC development.

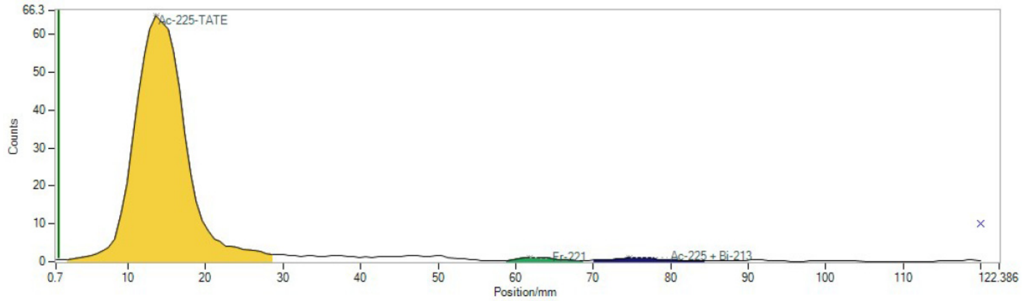

Figure S5: Analytical radio-TLC of ^{225}Ac-TATE after purification using a CM cartridge (0.1 M citrate pH 5.0 on silica gel-aluminum F254). RCP >97% 1 h past TLC development.

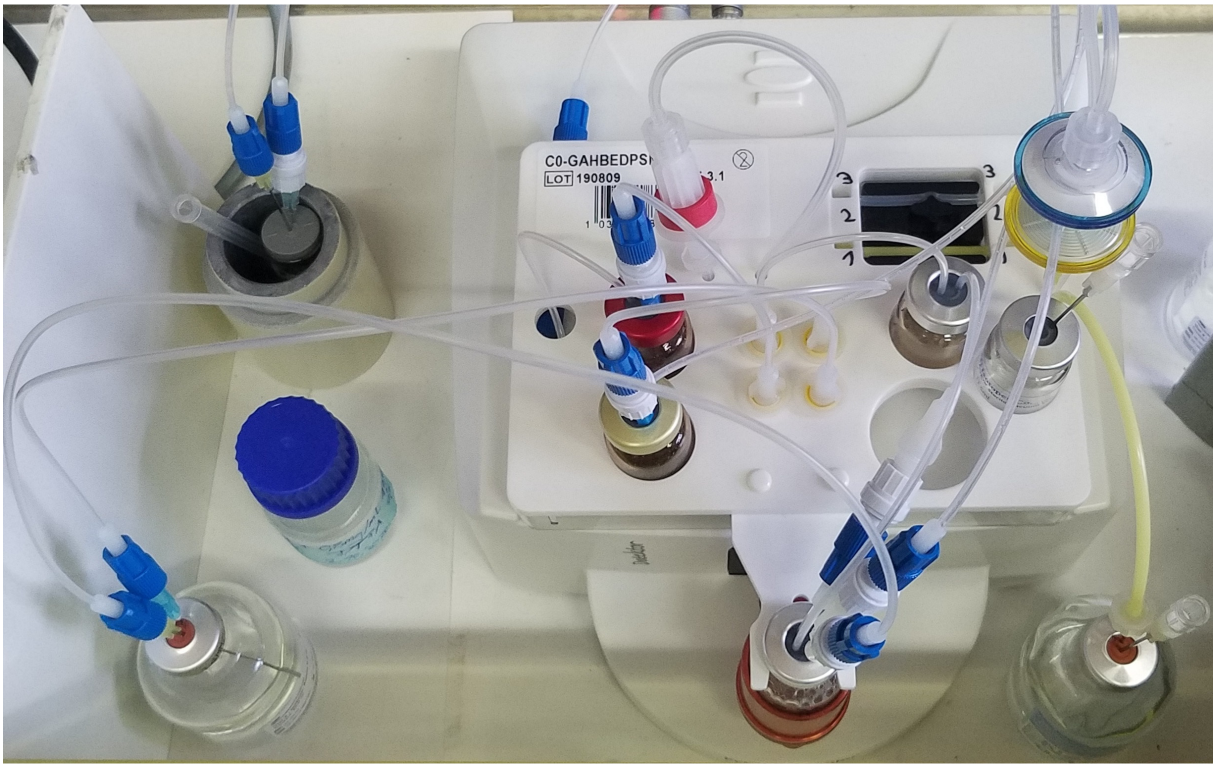

Figure S6: Modular-Lab EAZY module in its final configuration for C18 purification. At the upper left is the KIMAX vial with the activity. The saline in on the bottom left. At the bottom in the middle is the reactor, at the bottom on the right is the waste vial, and on the right side with filters is the product vial. In the center are the buffer vial with the gold cap and the eluent vial with the red cap.

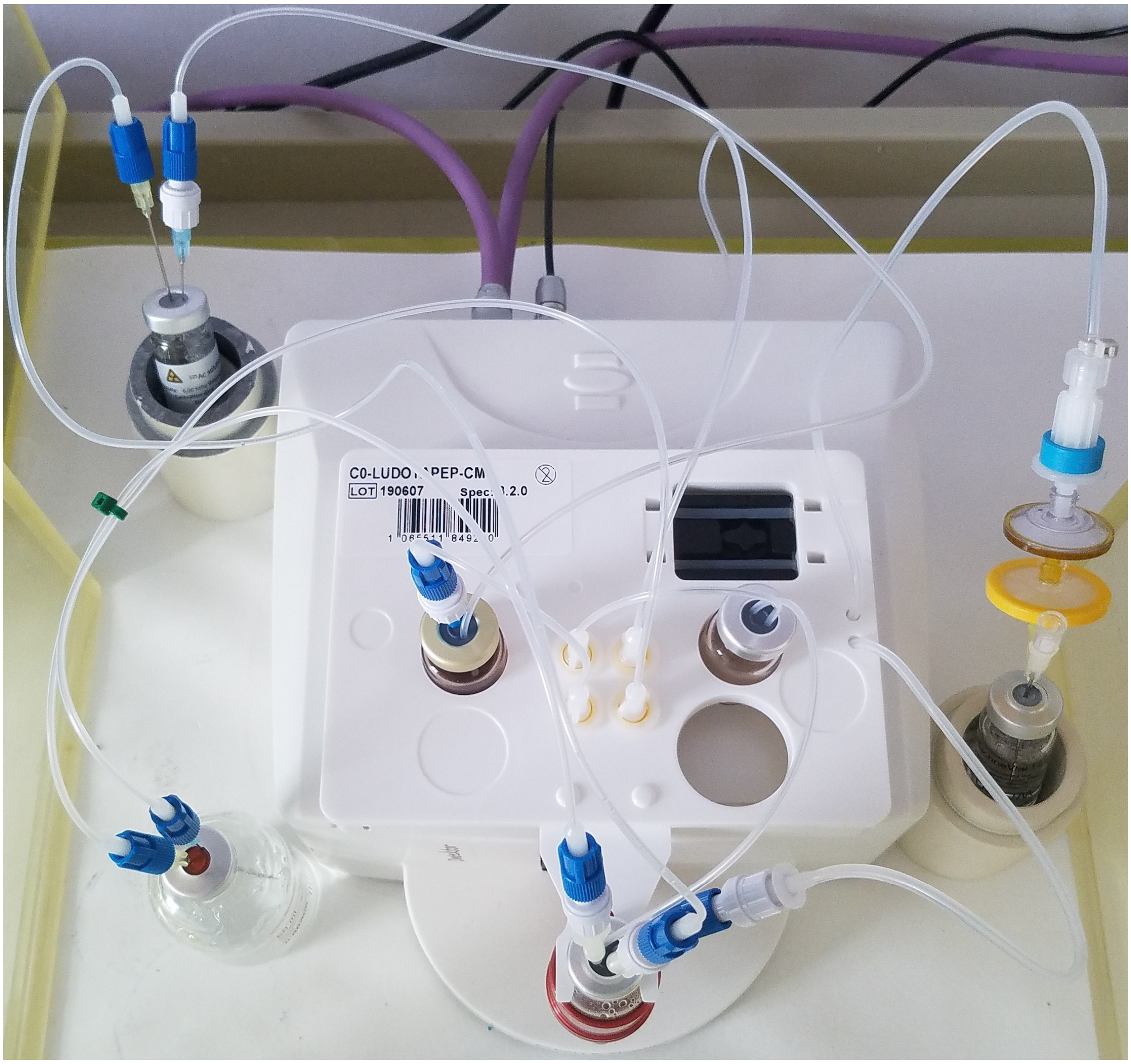

FIGURE S7: Modular-Lab EAZY module in its final configuration for CM purification. At the upper left is the KIMAX vial with the activity. The saline in one the bottom left. The reactor is at the bottom in the middle and the product vial with the filter is on the far right. In the center towards the left is the buffer vial with the gold cap.

Table S1. Overview of ^{225}Ac-syntheses on ML EAZY

											measurements of TLC-strips with CoMo-170 (IPS)						
											silicagel-aluminum citrate buffer 0.1 M pH 5			ITLC-SA 1 M NH_4Ac:MeOH 1:1			
**B.#**	**A[a] (MBq) HCl[b] (mL) Ac-salt**	**pre-cursor (µg)**	**A-C18 (MBq)**	**A-filter (MBq)**	**A-CM (MBq)**	**A-prod (MBq)**	**A-waste (MBq)**	**pH prod**	**endo-toxin <5 EU /mL**	**RCY**	**origin**	**front**	**RCP**	**origin**	**front**	**RCP**	**comments/ changes to the prior syntheses**
	1.8	TATE															reaction 35 min at 90°C
01	0.20	40	0.10	0.10		**0.37**	1.16		n.d.	**6%**	12	28	30%	4	42	91%	eluent 1.5 ml 50% EtOH + 2 mg
	NO_3^-					**0.12**	1.16				5	31	14%	4	56	7%	DTPA
	1.3	TATE															
02	0.20	40				**0.16**	1.05		n.d.	**13%**	9	34	21%	5	48		pressure program adjusted
	NO_3^-										15	21	41%	5	75	6%	
	2.4	TATE															
02b	0.20	40	0.20		0.33	**0.39**	2.01		n.d.	n.d.	60	76		44	137		pressure program adjusted
	NO_3^-										9	28		13	48		
	2.9	TATE															>10% activity left in two filter
03	0.10	120	0.10	0.30		**2.10**	0.30	5.6	yes	**75%**							1x MILIEX GS (vented) plus
	NO_3^-					**2.20**	0.04				1059	8	>99%	10	1430	>99%	1x MILLEX GV (unvented)
	4.1	TATE															
04	0.15	120	0.04	0.30		**3.40**	0.60	6.1	yes	**87%**	515	66	89%	18	543	97%	stable method C18
	NO_3^-					**3.59**	0.04				1402	4	>99%	10	1825	>99%	
	5.1	TATE															
05	0.2	160	0.10	0.50		**4.35**	0.20	6.2	yes	**86%**	331	22	94%	5	228	97%	
	NO_3^-										779	1	>99%	7	773	>99%	
	9.7	TATE															
06	0.40	240	0.20	0.80		**8.12**	1.50	6.0	yes	**83%**	239	17	93%	8	303	97%	
	NO_3^-										495	1	>99%	4	894	>99%	
	4.9	TATE															
07	0.10	60	0.04	0.40		**3.34**	0.20	6.0	n.d.	**69%**	295	12	96%	8	243	97%	
	Cl^-			0.30		**3.53**				**72%**	300	31	91%	9	316	97%	
07[f]											605	1	>99%	3	924	>99%	product stability after 2 h
											648	20	97%	3	727	>99%	
	5.8	TATE															
08	0.10	100	0.10	0.30		**4.68**	0.70	5.6	n.d.	**80%**	285	25	92%	6	335	98%	<5% activity left in two filter

	Cl^-											>99%			>99%	2x MILLEX GV (vented and unvented)
	6.2	TATE														reaction now at 105°C
09[g]	0.14	120	0.04	0.20	**5.25**	0.20	4.3	n.d.	**84%**	1120	380	75%	70	1410	95%	2x MILLEX GV (vented and
	NO_3^-									2000	70	97%	35	1830	98%	unvented)
	3.0	TATE														
10	0.14	60	0.04	0.04	**2.20**		4.6	n.d.	**90%**	541	109	83%	32	205	86%	automated C18-conditioning programmed
	NO_3^-				**2.70**	0.10										
	3.4	PSMA														
11	0.15	50	0.04	0.10	**2.50**	0.70	4.6	n.d.	**74%**	117	8	95%	25	244	91%	
	NO_3^-					0.40				235	1		35	592		
	3.2	PSMA														
12	0.15	25	0.10	0.20	**2.10**	0.80	4.7	n.d.	**65%**	75	16	82%	36	122	77%	to less precursor/MBq
	NO_3^-				**1.90**					226	26	90%	98	250	72%	
	1.7	PSMA														
13	0.15	25	0.20	0.20	**1.40**	0.40	4.6	n.d.	**82%**	100	5	95%	23	140	86%	
	NO_3^-				**1.30**	0.04				262	3	99%	55	347	86%	
	3.3	PSMA														First cassette prototype directly
14	0.20	50	0.10	0.04	**2.40**	0.60	4.6	n.d.	**73%**	112	12	90%	45	175	79%	obtained from E&Z #200828.
	NO_3^-				**2.30**	0.40				300	7	98%	75	375	83%	irradiated C18 used
	5.4	TATE														
15	0.10	50	0.10	0.10	**4.20**	1.20	4.6	n.d.	**78%**	327	6	98%	7	230	97%	pressure programming adjusted for irradiated C18
	Cl^-				**4.10**					954	5	99%	5	1019	99%	
	16.9	TATE														
16	0.30	340	0.20	0.40	**15.40**	1.40	4.5	n.d.	**91%**	1040	53	95%	22	778	97%	3x patient dose ^{225}Ac-TATE
	NO_3^-				**15.00**	0.30				2413	25	99%	11	2568	99%	
	17.9	PSMA														
17	0.30	320	0.10	0.70	**15.40**	1.30	4.4	n.d.	**86%**	962	143	87%	483	1799	79%	3x patient dose ^{225}Ac-PSMA
	NO_3^-									2758	49	99%	707	2945	81%	
	4.5	PSMA														
18	0.10	60	0.10	0.04	**2.80**	1.10	4.7	n.d.	**62%**	700	300	60%	100	470	82%	pressure programming adjusted to less precursor/MBq
	NO_3^-									580	7	99%	200	900	82%	
	4.3	PSMA														
19	0.10	120	0.04	0.20	**3.30**	0.50	4.7	n.d.	**79%**	870	85	88%	100	960	90%	double amount precursor/MBq

	NO_3^-					**3.40**	0.50				2230	17	99%	225	2270	91%	
	2.8	PSMA															
20	0.10	60	0.04	0.10		**2.20**	0.30	4.8	n.d.	**82%**	290	37	87%	9	470	98%	stable method
	NO_3^-					**2.30**	0.30				908	1	99%	24	940	98%	
	5.2	PSMA															
21	0.10	110	0.04	0.10		**4.40**	0.30	4.8	yes	**80%**	290	37	87%	9	470	98%	first patient dose
	Cl^-										908	1	99%	24	940	98%	
	5.5	PSMA															
22	0.10	120	<0.04			**4.90**	0.30	4.6	yes	**89%**	500	30	94%	100	665	86%	second patient dose
	Cl^-			0.10							1495	1	>99%	319	1195	79%	
	5.3	PSMA															
23	0.10	110	<0.04			**3.30**	0.90	4.1	yes	**88%**	870	33	96%	5	1200	99%	third patient dose
	Cl^-			<0.04							2200	22	99%	50	2260	98%	
	5.1	PSMA															fourth patient dose
24	0.40	110	<0.04			**4.50**	0.60	5.9	yes	**88%**	226	17	93%	25	259	91%	no DTPA in final solution
	NO_3^-			<0.04			0.40				631	15	98%	17	676	98%	
	3.4	TATE															
25	0.10	60		0.10	<0.04	**2.80**		6.1	n.d.	**82%**	311	51	86%	83	208	71%	CM-cartridge and LuPep-cassette stability after 18 h >94%
	NO_3^-										591	38	94%	7	1036	99%	
	3.1	PSMA															
26	0.10	60		0.10	0.10	**3.00**		6.0	n.d.	**97%**	385	28	93%	87	619	88%	CM-cartridge and LuPep-cassette stability after 18 h >90%
	NO_3^-										891	28	99%	49	1217	96%	
	1.2	TATE															1.2 MBq ^{225}Ac were added to the
27	0.15	20		0.10	1.10	**1.10**		6.1	n.d.	**58%**	142	11	93%	12	96	89%	reactor after dilution with saline
	NO_3^-			<0.04	1.50	**0.70**					330	11	97%	11	307	97%	for testing of CM
	5.9	PSMA															stable CM-method
28	0.10	60		0.30	0.10	**5.30**		6.2	yes	**90%**	335	17	95%	60	1048	95%	fifth patient dose
	Cl^-										755	7	99%	16	2065	99%	
	6.0	PSMA															sixth patient dose
29	0.10	120		0.10	0.10	**4.90**		5.5	yes	**82%**							TLC-scanner used for quality
	Cl^-												>99%			95%	control
	6.0	TATE															
30	0.10	120		0.10	0.20	**4.70**		6.2	yes	**78%**							seventh patient dose
	Cl^-												89%			96%	

[a]A stands for Activity

[b]HCl was always 0.04 M

[c]buffer was always 2 mL NaAc/AcOH 0.1 M from -20°C

[d]reaction pH was always between 5.0–5.5

[e]the second value for activity in on column is always measured 2 h later

[f]TLC stability test 2 h after syntheses

[g]reaction temperature was changed from this batch on for all following batches from 90°C to 105°C

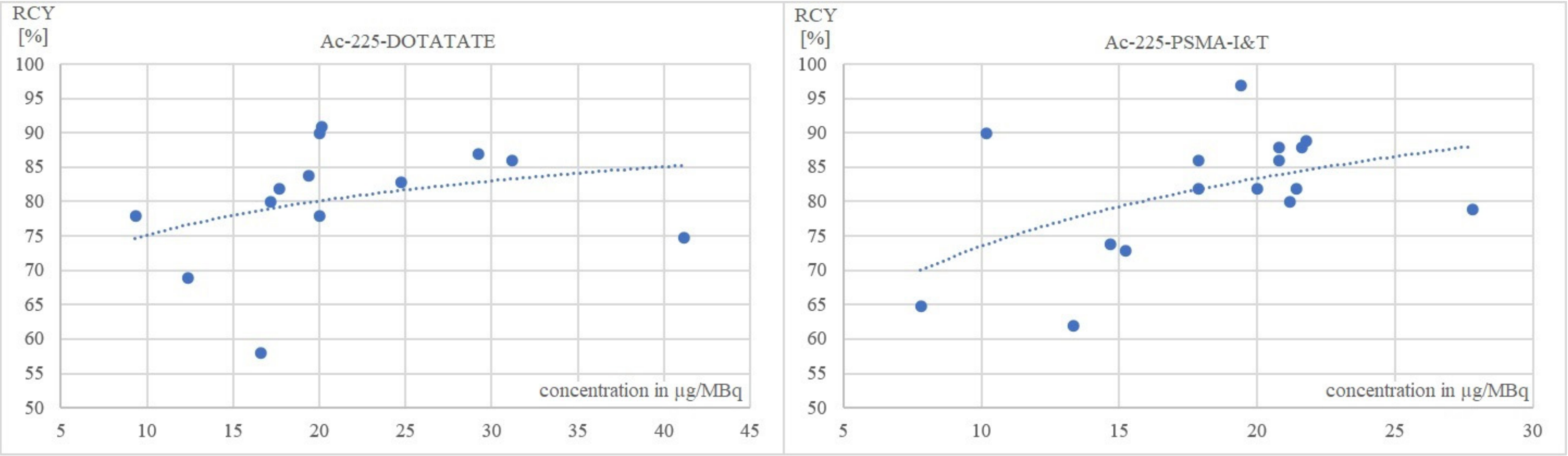

Figure S8: Graphical illustration for table S1 of radiochemical yield (RCY) versus precursor amount per activity for ^{225}Ac-DOTATATE (n = 13) and for ^{225}Ac-PSMA-I&T (n = 16) after Modular-Lab EAZY module syntheses.

Table S2. Formation of daughter nuclides of ^{225}Ac to quasi-stable ^{209}Bi and stable ^{205}Tl

**nuclide**	**possibility**	**half-life**	**modes of decay**	**energy**
^{225}Ac	~100 %	9.92 d	α (100%)<br>γ (3% co-emission)	5.8 MeV<br>60–100 (78) keV
^{221}Ra	<0.000001 %	28 s	α (100%)	6.9 MeV
^{221}Fr	100 %	4.8 min	α (100%)<br>γ (12% co-emission)	6.3 MeV<br>218 keV
^{217}Rn	0.007 %	0.54 ms	α (100%)	7.9 MeV
^{217}At	100 %	32.3 ms	α (100%)	7.1 MeV
^{213}Po	97.800154 %	3.7 µs	α (100%)	8.4 MeV
^{213}Bi	99.993 %	46 min	α (2%)<br>β (97.8%)<br>γ (26% co-emission)	5.9 MeV<br>1.4 MeV<br>440 keV
^{209}Pb	100 %	3.25 h	β	0.6 MeV
^{209}Tl	2.199846 %	2.16 min	β	2.0 MeV
^{209}Bi	100 %	1.9×10^{19} a	α	3.1 MeV
^{205}Tl	100 %	∞		
